# Parental Acceptance of HPV Vaccine in Peru: A Decision Framework

**DOI:** 10.1371/journal.pone.0048017

**Published:** 2012-10-29

**Authors:** Rosario M. Bartolini, Jennifer L. Winkler, Mary E. Penny, D. Scott LaMontagne

**Affiliations:** 1 Instituto de Investigación Nutricional, La Molina, Lima, Peru; 2 PATH, Seattle, Washington, United States of America; 3 Department of Global Health, University of Washington, Harborview Medical Center, Seattle, Washington, United States of America; IPO, Inst Port Oncology, Portugal

## Abstract

**Objective and Method:**

Cervical cancer is the third most common cancer affecting women worldwide and it is an important cause of death, especially in developing countries. Cervical cancer is caused by human papillomavirus (HPV) and can be prevented by HPV vaccine. The challenge is to expand vaccine availability to countries where it is most needed. In 2008 Peru’s Ministry of Health implemented a demonstration project involving 5^th^ grade girls in primary schools in the Piura region. We designed and conducted a qualitative study of the decision-making process among parents of girls, and developed a conceptual model describing the process of HPV vaccine acceptance.

**Results:**

We found a nonlinear HPV decision-making process that evolved over time. Initially, the vaccine’s newness, the requirement of written consent, and provision of information were important. If information was sufficient and provided by credible sources, many parents accepted the vaccine. Later, after obtaining additional information from teachers, health personnel, and other trusted sources, more parents accepted vaccination. An understanding of the issues surrounding the vaccine developed, parents overcome fears and rumors, and engaged in family negotiations–including hearing the girl’s voice in the decision-making process. The concept of prevention (cancer as danger, future health, and trust in vaccines) combined with pragmatic factors (no cost, available at school) and the credibility of the offer (information in the media, recommendation of respected authority figure) were central to motivations that led parents to decide to vaccinate their daughters. A lack of confidence in the health system was the primary inhibitor of vaccine acceptance.

**Conclusions:**

Health personnel and teachers are credible sources of information and can provide important support to HPV vaccination campaigns.

## Introduction

Cancer of the cervis is the third most common cancer affecting women worldwide. Cervical c is preventable but continues to cause the deaths of more than 270,000 women worldwide each year [1], of whom over 85 percent live in developing countries where existing programs to detect and provide timely treatment do not reach or are beyond the means of most women [1], [2]. Each year in Peru, cancer of the cervix is responsible for the deaths of an estimated 2,098 women [1] and is the most common cause of mortality among women 25 to 44 years old [3].

Two strains of human papillomavirus (HPV), types 16 and 18, account for about 70 percent of cervical cancers [4], approximately 90 percent of anal cancers, and a smaller subset (<50 percent) of other cancers, e.g., oropharyngeal, penile, vaginal, and vulvar [5]. Vaccines against the two most common HPV types, 16 and 18, have proven safe and efficacious [6], [7] in preventing precancerous lesions in HPV-naive girls and women. Prophylactic vaccination targeting these genotypes is expected to result in significant reductions in the burden of cervical cancer and other cancers associated with these genotypes, provided that these vaccination programs can achieve significant coverage of the target population [8].

New vaccine adoption has taken more time in lower-resource settings: hepatitis B virus vaccine adoption in low-income countries took nearly 20 years, twice as long as in high-income countries [9]. Vaccine price is often a key factor in vaccine decision-making [10], though this may be less true for countries eligible for subsidized vaccine through the GAVI Alliance. Prior to widespread HPV vaccine introduction, speculation about potential significant adoption barriers focused on several issues: the target age group was outside the routine infant immunization schedule, the vaccine was for girls only, the vaccine protected against a sexually transmitted virus, and the benefits of vaccination were long term rather than immediate [11], [12].

Peru’s Ministry of Health implemented an HPV vaccine demonstration project to study the issues necessary to make informed decisions about the introduction of the vaccine into the national immunization strategy. This project implemented HPV vaccination in 2008 to girls aged nine years or older in grade 5 of state and private primary schools in a predefined area of the region of Piura that included rural, urban, and peri-urban locations. The project used existing health and education systems and structures at local and regional levels for community sensitization and mobilization, vaccine administration, delivery, and cold chain maintenance, and monitoring and supervision [13]. The immunization program in Peru is well established and nearly universally recognized at the community level.

School-based vaccination programs for HPV may bring additional challenges including informing parents and girls and coordinating with the educational system, particularly teachers. The dynamic between opportunity, information, authorization, and informed consent for HPV vaccination in schools is a balance that depends on many circumstances, and one that has been under-investigated in studies to date [14]. To explore this dynamic, we studied the decision-making process among parents of girls eligible for HPV vaccination in Peru and developed a conceptual model describing the process of vaccine acceptance.

## Methods

We designed a qualitative descriptive study of HPV vaccine acceptability and decision-making among parents of girls eligible for vaccination. We selected schools that would capture the diversity of situations at the school level within the area that implemented HPV vaccinations.

### Ethics Statement

We obtained verbal consent from all parents who agreed to be interviewed for this study. Researchers read the scripted verbal consent exactly as written so that the process was standardized for all persons invited to participate in the study. Research staff signed all verbal consent forms after verbal consent was obtained to document the completed process and agreement of individual parents to participate. Transcribed tape recordings of each interview were assigned a unique identifier to maintain confidentiality. All data were kept in secure files, and computerized records were password protected with access limited to research staff.

This study and its ethics procedures were approved by the research ethics committees of PATH in the United States and the Instituto de Investigación Nutricional in Peru.

### Sampling Process and Participants

In close collaboration with regional ministry of health staff in charge of immunization, we selected 12 schools–six in urban areas and six in rural areas–where HPV vaccination had been carried out (Table 1). The goal was to represent diversity not only in rural and urban populations, but also in factors such as affiliation with health facilities, high and low coverage of HPV vaccine at first dose (as a surrogate measure of successful programs and those that experienced challenges), when HPV vaccination was first introduced, and size of the affiliated health facility (including hospitals).

**Table 1:** Criteria used in the selection of health facilities in Piura.

Health network	Health facility	Urban	HPV vaccination 2007–2008	HPV vaccination introduced in 2008	High coverage	Low coverage
RED Bajo Piura	CS Catacaos	•				•
	CS Bernal			•		
	CS La Legua*					
RED Chulucanas Morropón	Hospital Chulucanas	•	•			
	CS Morropón	•	•			
	CS Buenos Aires				•	
	CS Yapatera				•	
	CS Chalaco				•	
RED Huarmaca	CS Huarmaca			•	•	
RED Piura Castilla	CS Pachitea	•	•		•	
	Hospital Sta. Rosa	•	•			
	CS San José	•	•			•

* Note: CS La Legua was selected based on its rural location.

Within each of the 12 participating schools, we selected and interviewed parents of two girls who received all three doses of HPV vaccinations and parents of two girls who were not vaccinated with HPV vaccine. The total sample size was 48 parents. We asked teachers to suggest parents who were likely to collaborate and share their experience of acceptance or refusal of the vaccine. They received invitations inviting them to participate in the study.

### Data Collection, Management, and Analysis

In-depth interviews were conducted with each parent by qualitative researchers with experience in anthropological interview methods. The guided interview covered the HPV vaccination program, educational and promotional materials and activities, method of learning about the program, opinion about the implementation of the vaccination program, factors that influenced acceptance (or nonacceptance), and suggestions for program improvements.

The in-depth interviews were recorded and then transcribed into thematic matrixes generally retaining the textual expression of the interviewee. Each thematic matrix was considered in relation to the others to develop an integrated idea of the conditions and factors that dealt with the parents’ acceptance and nonacceptance of the HPV vaccine. This data analysis involved the reconstruction or understanding of the points of view of the parents, identifying the differences, similarities, and patterns within urban and rural environments. We separately analyzed the data that supported acceptance of the vaccine and the decision-making process and the data related to nonacceptance. We used quotations to reinforce the data analyzed and developed a conceptual image to summarize our main findings [15].

## Results

### Dynamic Decision-making Process for HPV Vaccine

We observed a nonlinear decision-making process among parents that evolved over time. The decision-making was influenced by the context–particularly the way in which vaccination was offered, the follow-up by the health personnel, the commitment shown by the teaching staff, and the inter-relatedness of these elements. The parents and the girl made the decision, which was influenced by others.

**Figure 1:**
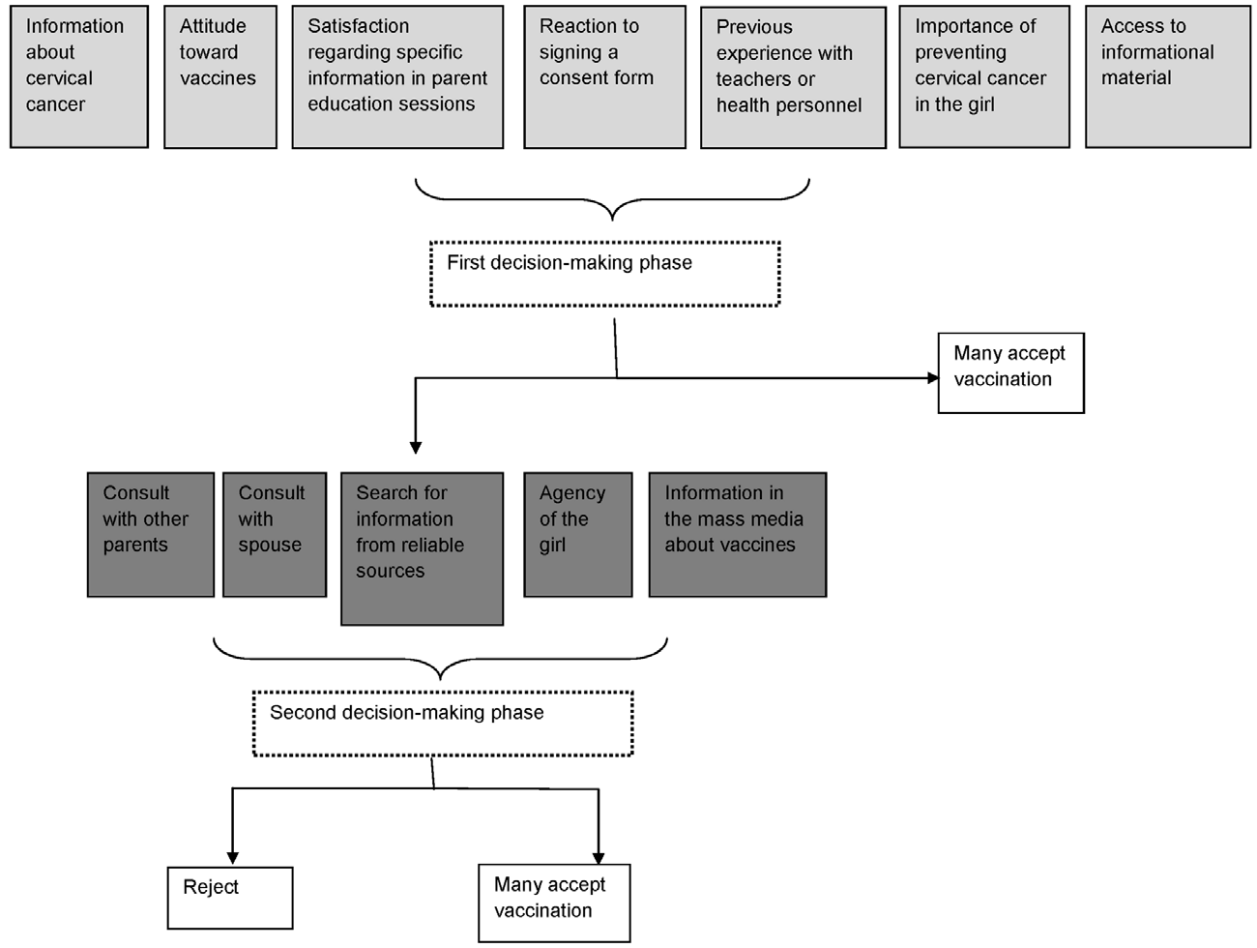
Conceptual model for parental decision-making for HPV vaccine in Piura. The figure illustrates how a variety of different perceptions, experiences, knowledge, and attitudes provide a background context and influence a mother and/or father’s decision to vaccinate their daughter. Divided into phases, the decision-making model demonstrates that if the basis of this decision is sufficiently positive, parents may proceed to accept vaccination; however, if doubts remain, parents may seek further information or opinions and may modify their decision, crystallizing it into refusal or acceptance. Model tested in northern Peru.

We identified at least two phases in this process: the first reaction and preliminary decision, and the second phase during which others influenced the final decision (Figure 1). These phases were relevant for both parents who accepted and those who did not accept HPV vaccine for their daughters. In the first phase, the newness of the vaccine and the unusual requirement of written consent from the parents were of particular importance. In the face of a new vaccine and a new modality of providing a vaccine (only to girls in grade 5 and with a signed informed consent), the process of decision-making signified a process of acquiring confidence in the midst of rumors and negative comments about the vaccines. The information that parents received about the disease and the vaccine allowed for a preliminary positioning, but in many instances was not sufficient for making a decision. If the information provided early-on, specifically to parents, was sufficient, and if those who provided the information (teachers or health personnel) had good credibility, many parents accepted the vaccination. This was particularly true in rural areas, and in urban schools with a positive previous experience between parents and teachers or between health personnel and parents.

In the second phase, the activities implemented by teachers and health personnel and information parents and girls found from other sources allowed them to change their minds. It was a process of developing a better understanding of the issues surrounding the vaccine and cervical cancer, and overcoming fears, rumors, and internal negotiation within the family. Nonetheless, many parents looked for additional information about the vaccine through avenues in which they had more confidence, or looked for the agreement of the other parent. The girl’s own perspective also played a role and generated a second phase in the decision-making, particularly for families in urban areas.

### Factors that Favored Acceptance of HPV Vaccine

#### Community sensitization meetings with parents

Immediately following meetings on cervical cancer and the HPV vaccine, many parents agreed to give their consent. This was true in both urban and rural areas, but particularly pronounced in rural areas. Following what they heard in the educational session or read in the informed consent and reflected on at home, they felt that these meetings helped them understand the issues, allowed them to ask questions, and encouraged them to accept the vaccination. Nonetheless, afterwards they also asked other people about their perspectives and talked to their husband or wife. Some mentioned that they made a decision after the informational meeting and felt a meeting like that should always be offered. Some parents heard about the vaccine for the first time at this meeting. Some parents said that their daughters talked to them about what they had been taught about cervical cancer and the HPV vaccine.

#### Vaccines are a well-recognized and accepted form of prevention

The parents who accepted HPV vaccination said they knew vaccines help prevent or cure illnesses, are given to children, and represent financial savings for the family because the children do not get those illnesses. Since families do not need to invest in treating the associated illness, vaccines are considered desirable for families with limited economic resources.

I think vaccines are good. If it’s a question of saving lives, then the vaccine is welcome. I always support having my daughters vaccinated. Right from the start I accepted it. As I said before, I always have my daughters vaccinated because it protects life. (urban mother)

#### HPV vaccine can prevent cervical cancer, a serious illness

The parents who accepted the HPV vaccine also agreed that cervical cancer is a frequent, serious, and deadly illness, and that it causes a lot of suffering for women who develop it. They also commented that treatment is costly and treatment services either do not exist in the region or are not available to all women. Those interviewed often described cases they knew personally, which made it even more important to them to accept a preventative measure against this illness.

… and also because she benefited as well, due to the illnesses, the cancer that’s currently affecting a lot of people… it’s really advanced. There’s been an increase in cases of cervical cancer. There are more cases than before and the number is growing every day. So the need to protect her made me see that the vaccine was a good thing. (rural mother)

#### Teachers influenced the environment of decision-making

Many parents also said they trusted the teacher, the school, and the health personnel, arguing that if they had approved the vaccination at the school then it was a good thing for their daughters; this assessment was particularly true in rural areas. Some parents stressed that they trusted the teachers at their schools. Other parents responded to the advice given by the school head teacher or administrative staff. Parents generally emphasized the long experience of trust they had with these people and institutions over the years. In some settings, however, parents described schools where the teachers were not respected or the parents always opposed what the teachers told them.

I had not heard of the vaccine for the cervix. For my part I was afraid that it was going to be a dangerous thing because sometimes they get vaccinated and sometimes they die, they become ill or die. And that is the fear that I tell you about as a mom, I was afraid to have them give her the vaccine. So we did not want to accept it. Between us we wondered if it would give a good result. We were so hesitant. The teacher told us not to be afraid as the cervical cancer vaccine is important to them. And we let ourselves be persuaded by the teacher. (rural mother)

#### HPV vaccines are expensive, so we should take advantage of the free opportunity

Many parents, especially those in urban areas, mentioned that they decided to accept HPV vaccination because it was being given free to 5^th^ grade girls. Since the vaccine was too expensive for them to afford through the private sector, they did not want to miss this chance. Parents learned about this opportunity from teachers, health personnel, and their daughters.

Yes, they announced that it was a privilege to have the pilot project start in the city, because the vaccine was very expensive and they were giving it in areas with economic shortages… Apart from the information they gave about a better future, it was due to the part that they were helping the population’s health by giving something that was so expensive, making it free… and, well, if it doesn’t cost anything for people with no resources, (you have to) take advantage.” (urban mother)

#### Positive media reports about HPV vaccines

A multi-level communication campaign was implemented in Piura. Local communication strategies varied across the region. In one of the rural mountainous zones, extensive dissemination about the HPV vaccine was done through the municipality’s radio station and through contacts with the local Catholic Church. At the mass-media level, the press and television maintained attention on the campaign through separate announcements of each of the three doses of the HPV vaccine and regional news briefs. Some girls and mothers reported having heard or seen news items on television. Also at the mass-media level, campaign posters and banners were displayed on the front of the health facilities and some schools. Many mothers and girls mentioned having seen the banners, which reassured them about the official nature of the vaccination event.

#### Other parents, relatives, and health personnel were supportive

After the informational meetings at schools, particularly in the urban areas, most parents discussed their thoughts and doubts about the vaccine within their family and with other parents. They also looked for additional information on the Internet or sought medical advice from health professionals. Only after they received a favorable opinion about the HPV vaccine from this additional information did they agreed to vaccinate their daughters.

#### Decision to vaccinate involved both parents

In both urban and rural areas some mothers wanted to talk to their husbands about the decision, even after they had decided that they wanted to vaccinate their daughters. Some of these mothers described their relationship with their husbands as one of trust and communication, in which the husband trusted what she decided. Other mothers explained the decision to vaccinate their daughter was one they would make jointly with their husbands as it was of particular importance.

They gave us a piece of paper to sign and you had to tell your husband about it. I explained to my husband what they told us in the talk and we agreed to have her vaccinated… he said it was OK for her to be vaccinated. If your husband didn’t agree? They didn’t give her the vaccine, because if something happened to her it was my responsibility; you both had to agree.” (rural mother)

The fathers interviewed said that they heard about the importance of the vaccination on their daughter’s future health from their wife or daughter. Some fathers recommended that the mother consult the teacher or health worker again just to be sure, while others agreed with their wife or daughter’s inclination to vaccinate. Some fathers also mentioned that because this was a women’s health issue, it was more appropriate for mothers to make this decision.

…I talked to my husband (…) the first thing he said to me was, “Ask your sister to ask the doctors, to find out, because I don’t know, I don’t really understand…” (urban mother)

#### Educational materials

For some parents, particularly in urban areas, the educational leaflet distributed prior to vaccinations provided important information about the vaccine and helped them make their decision. They remembered the contents of the leaflet described the purpose of the vaccine and recalled how the illustrations included had explained the illness.

#### The influence of the girls eligible for vaccination

Some mothers in urban areas said that it was their daughter who convinced them to get the vaccine. Some daughters asked for vaccination, in one case crying, concerned that she would get sick or even die if she was not vaccinated.

Yes, she wanted to be vaccinated, and on top of that she’s thin and told me the vaccine would surely make her put on weight. She also said, “They’ve already vaccinated me against hepatitis B and nothing happened to me; so, mom, let me be vaccinated.” She’s not scared of vaccines. (urban mother)

### Factors for Non-acceptance of HPV Vaccine

#### Vaccine side effects

Some parents in both urban and rural areas believed that a disease as serious as cervical cancer would require an equally strong vaccine, and were concerned that a vaccine of this strength could harm their daughters. Many parents who did not accept HPV vaccine feared the vaccine would cause sterilization or affect the normal development of the female reproductive organs.

I was scared because she still isn’t menstruating. I said perhaps it’s going to affect her menstruation. And I heard somewhere that you end up sterile after having that vaccine. (urban mother)

All of us moms said no because of the rumors about sterilization, or the effects after applying vaccines, because there was a rumor at the time about the hepatitis vaccine, even that children had died because of the vaccine. So that frightened us. (urban mother)

#### Consent for vaccination as a barrier

Some parents, particularly those in urban areas, felt that signing an authorization for their daughter to be vaccinated meant accepting responsibility for any negative reaction to the vaccine. This consent process generated distrust as consent had not been requested for other vaccines.

#### The influence of the girl eligible for vaccination

In some cases the parents wanted to vaccinate their daughter, but she did not want to be vaccinated, claiming that it hurt a great deal. Some parents mentioned that they did not insist on vaccination for this reason.

My daughter did not want to be vaccinated, said flatly no. And so daughter if you do not want it, I won’t force you. For that reason I did not… (urban mother)

#### Absence of information about the HPV vaccine in the mass media

Parents mentioned that a lack of information about the HPV vaccine in the media increased their distrust as they felt the vaccine campaign might be an experiment that was being hidden or kept secret.

For the hepatitis B (vaccine) … we knew about it from the media. We haven’t had complete knowledge about the uterine cancer (vaccine) because we have seen the media and there has been no information … I do not know where the vaccine came from. The Ministry of Health always provides information and in this case there wasn’t any … My husband told me that they had told him they wanted to sterilize girls.(He said)“How do you know that these vaccines are really for uterine cancer, or is it for something else?” (urban mother)

On the other hand, parents reported that they heard news related to problems with other vaccines. In coastal urban and rural zones in particular, parents mentioned in interviews that the decision-making process was influenced by news stories related to cases of vaccine-related death due to yellow fever or measles/rubella vaccines and by news of expired vaccines in the area’s health facilities. These reports generated a general fear of vaccinating their daughters, and increasing distrust of the HPV vaccine among parents.

We heard the news of a child who had been vaccinated in Lima against hepatitis B and lost her ability to speak. So my husband was afraid to vaccinate my daughter. (rural mother)

#### The role of fathers in authorizing health care for something serious

Given the uncertainty and fears surrounding the vaccine, some mothers mentioned that they left the decision in the hands of the girl’s father. In some cases, the mother did not want the responsibility of making the decision about her daughter’s vaccination, even when she herself wanted her daughter to be vaccinated.

Her dad had to give the order. If her dad said yes, I said yes, too. If he says no and I say yes, suppose something happened to the baby. That’s why. (rural mother)

#### Vaccine may promote sexual promiscuity

In just one family interviewed, one parent argued that the HPV vaccine would encourage their daughter to have sexual relations and would have a negative effect on her health.

Her dad didn’t want to authorize it because he said it encourages having sexual relations with anyone. I explained to him that it was a vaccine to protect her against cervical cancer, but he didn’t want to sign. He was also afraid something might happen to her. (rural mother)

#### Limited or unclear information

Some parents mentioned that they did not have enough information to make the decision about vaccination, or felt the information they had received was not sufficient. They cited not having any written material about the HPV vaccine. Some said that they did not go to the educational meeting for parents, but also did not receive other information on the subject–or only received information through their daughter. This was noted more commonly in urban than in rural settings.

## Discussion

The broader concept of prevention (cancer as a danger to be avoided, future health of daughters, avoiding risks, and vaccines as trusted strategy) combined with pragmatic factors (free, available at school) and the credibility of the offer (information in the media, recommendation of teacher or other respected authority figure) were central among the multiple motivations that led parents to choose to vaccinate their daughters against HPV. All these factors have emerged previously in the literature. Women from a rural area in North Carolina who associated negative consequences or reported high perceptions of cervical cancer risk were more accepting of the HPV vaccine [16]. Likewise a Swedish study identified a parent’s attitude toward vaccination in general as a correlate to their willingness to vaccinate their child against HPV [17]. In a study of maternal acceptance of the HPV vaccine in Malaysia, 98 percent of mothers said they would accept the vaccine for their daughters if it was provided routinely by the government [18]. Our findings of reasons for vaccination are also confirmed from a quantitative study of HPV vaccine coverage in Peru, India, Uganda, and Vietnam. More than two-thirds of parents of fully vaccinated girls indicated that they had their daughters vaccinated primarily for protection against cervical cancer, or because they believed that vaccines are good for health [19]. The HPV vaccine’s benefit for the prevention of cervical cancer has been noted in studies done in the United Kingdom and Australia, after they introduce HPV vaccine as a part of the national immunization program [20], [21].

Parents in our study also emphasized the importance of information and its provision through multiple trusted channels. Mass media was considered an important source of information about this and other vaccines, and lack of information about the HPV vaccine led to suspicion. Latina immigrants in a study of HPV vaccine in the United States reflected a similar sentiment in emphatically articulating that, to encourage others to get the HPV vaccine, more than one credible source of information would be needed [22]. Similarly, in a study of Salvadoran women and Latina women living in the United States, information was of the utmost importance. Among those who were unsure or would refuse the HPV vaccine, the most common reasons were “Because I don’t know enough about HPV” and “I want to talk to my child’s doctor first” [23].

We identified a lack of confidence in the health system as the primary inhibitor of vaccine acceptance. Some parents expressed this lack of confidence as fears about side effects such as sterilization, while others reported they did not have enough information to make a decision. These reasons are similar to those reported in a study of vaccine acceptance in the United Kingdom, where the main reason cited by parents was insufficient information about the vaccine and its long-term safety [20]. This finding parallels a survey of HPV coverage in Vietnam, where concerns about the safety of the vaccine and its possible experimental nature emerged, particularly in one urban location [19].

Concerns about vaccine safety have been increasing and other studies have revealed it is an important barrier to vaccination. A study of knowledge and attitudes about HPV and cervical cancer among adult women in northern Lima, Peru, found high levels of vaccine acceptability; however, worry about whether the vaccine was safe for use was an issue for 82 percent of respondents [24]. A study in Sweden found that adverse effects from the vaccine were the primary concern of Swedish parents in their unwillingness to vaccinate their children against HPV [17], which is similar to sentiments from Malaysian mothers [18].

While one parent cited fears of the vaccine causing early sexual activity, this issue was notably absent from most of the discourse of parents. A systematic review of the literature in 2007 identified four quantitative studies that addressed this issue; the authors found that only 6–12 percent of parents endorsed this concern [25]. Ferris and colleagues studied sexual disinhibition among 325 parents of children aged 9–17 years and found only 17 (5%) who thought that receiving HPV vaccine would encourage their child to have sex [26].

The HPV vaccine is different from most vaccines in the childhood vaccination schedule because it is recommended for girls aged 9−13 years, prior to sexual debut. However girls of this age often start expressing their own agency [27], an issue that is simply not part of the equation when parents are considering vaccinating infants. We observed that the HPV vaccine decision was not solely that of parents, but that the girl herself sometimes also influenced the parents’ decision–both in accepting and in rejecting the vaccine. In the previously cited study from the United Kingdom, 70 percent of girls interviewed stated that the vaccine decision was made jointly with her parents [28]. Studies from Australia [21] and Uganda [27] also found that adolescents have some independence in the decision to receive the HPV vaccine. Combined, these studies provide mounting evidence that decision-making for HPV vaccination has some degree of negotiation within the family, reflecting the maturation of adolescents and their increased independence from parents as they age.

Our conceptual model shares some core components with that developed from the work of Cooper Robbins and her colleagues [29] from the HPV vaccination program in Australia. Like us, they identified a framework in which some parents are decided and others are indecisive regarding whether to accept the HPV vaccine. They also identified the importance of parental confidence (or lack thereof) in the school as well as in the medical system. Their model differs from ours, however, in some key ways. Their model highlighted the individual family decision, whereas we observed that families in Peru reacted strongly in relation to the context of the provision of the vaccine. We identified the important contextual factor of teachers, health personnel, other parents, and the Internet in a central influential role–particularly in urban environments. Unlike the Australian model, we identified differences in the context of decision-making between urban and rural environments. In rural areas the parents were less decisive, while in urban areas they consulted with more people they trusted and took more time to come to a decision. In our model, the concerns of parents who did not accept vaccination were not as related to the sexual fate of their daughters (that it would encourage sexual depravity), but were rather about reproductive destiny: concerns about sterility or that the vaccine might be an experiment–essentially a core distrust of the government’s health system.

Lastly, we should note that the vaccine coverage achieved through this school-based HPV vaccination demonstration project in Piura, Peru, was quite high at 82.6 percent [19]. Indeed, vaccine coverage has been perceived to be the ultimate marker of acceptability, as a large proportion of the population agreeing to be vaccinated would signal broad support in the community. The preparations to implement the vaccination program in Piura could have influenced its feasibility [13], and in turn the acceptability to parents found in our study. The dynamic interplay between program preparations and community mobilization with program implementation and health worker competence has been noted in studies of the HPV vaccination program rolled out in Australia [29].

### Limitations

This study was limited in its relatively small sample size and geographic coverage of a single region within Peru. The study also took place in the context of a demonstration project rather than national implementation of the HPV vaccine. Some of the issues that emerged in this context may not be relevant to broad implementation of the vaccine. Nonetheless, the rich qualitative detail gives insights that may be relevant in a range of other settings, and the themes found in this study are echoed in quantitative data from other studies.

### Conclusions

This study identified a number of specific conditions that were important in the process of parental acceptance of the HPV vaccine in Peru, many of which may be relevant to other countries considering adoption of the vaccine.

Access to information is essential. Parents and girls themselves needed access to information that addressed their questions and allowed them to make informed decisions. Health personnel should be aware that vaccination of school-aged girls is not the same as infant vaccinations, in that girls this age are able to express their own opinions about vaccination and few vaccinations are delivered to children in this age group. Health personnel should be prepared to discuss these issues with parents and the girls. Teachers were also a trusted source of information for many parents, and programs may want to make sure that teachers understand the issues related to HPV vaccination and are prepared to share those details with parents and students.

Positioning of the HPV vaccine is important. Positioning the vaccine as an approach to combating cervical cancer strengthened parent confidence and helped parents to feel secure in their decision to improve the future health of their daughters. Programs will want to consider how to support parents in making good decisions for their daughter’s health. A focus on prevention may be useful in supporting parents in their decision-making.
